# Development and characterization of segment-specific enteroids from the pig small intestine in Matrigel and transwell inserts: insights into susceptibility to porcine epidemic diarrhea Virus

**DOI:** 10.3389/fimmu.2024.1451154

**Published:** 2024-09-17

**Authors:** Lu Yen, Rahul K. Nelli, Ning-Chieh Twu, Juan Carlos Mora-Díaz, Gino Castillo, Panchan Sitthicharoenchai, Luis G. Giménez-Lirola

**Affiliations:** ^1^ Department of Veterinary Diagnostic and Production Animal Medicine, College of Veterinary Medicine, Iowa State University, Ames, IA, United States; ^2^ Infectious Diseases Laboratory Research-LID, Facultad de Ciencia e Ingenieria, Universidad Peruana Cayetano Heredia, Lima, Peru; ^3^ Department of Population Health and Pathobiology, College of Veterinary Medicine, North Carolina State University, Raleigh, NC, United States

**Keywords:** porcine epidemic diarrhea virus, alphacoronavirus, infection, porcine enteroids, organoids, Matrigel, transwell culture, small intestine

## Abstract

**Introduction:**

The critical early stages of infection and innate immune responses to porcine epidemic diarrhea virus (PEDV) at the intestinal epithelium remain underexplored due to the limitations of traditional cell culture and animal models. This study aims to establish a porcine enteroid culture model to investigate potential differences in susceptibility to infection across segments of the porcine small intestine (duodenum, jejunum, and ileum).

**Methods:**

Intestinal crypt cells from nursery pigs were cultured in Matrigel to differentiate into porcine enteroid monolayer cultures (PEMCs). Following characterization, PEMCs were enzymatically dissociated and subcultured on transwell inserts (PETCs) for apical surface exposure and infection studies. Characterization of region-specific PEMCs and PETCs included assessment of morphology, proliferation, viability, and cellular phenotyping via immunohistochemistry/immunocytochemistry and gene expression analysis. Subsequently, PETCs were inoculated with 10^5^ TCID_50_ (50% tissue culture infectious dose)/mL of a high pathogenic PEDV non-S INDEL strain and incubated for 24 h. Infection outcomes were assessed by cytopathic effect, PEDV N protein expression (immunofluorescence assay, IFA), and PEDV N-gene detection (quantitative reverse transcription polymerase chain reaction, RT-qPCR).

**Results:**

No significant morphological and phenotypical differences were observed among PEMCs and PETCs across intestinal regions, resembling the porcine intestinal epithelium. Although PETCs established from different segments of the small intestine were susceptible to PEDV infection, jejunum-derived PETCs exhibited higher PEDV replication, confirmed by IFA and RT-qPCR.

**Discussion:**

This segment-specific enteroid culture model provides a reliable platform for virological studies, offering a controlled environment that overcomes the limitations of *in vivo* and traditional cell culture methods. Standardizing culture conditions and characterizing the model are essential for advancing enteroid-based infection models.

## 1 Introduction

Porcine epidemic diarrhea virus (PEDV) is an enveloped, single-stranded RNA virus under the order Nidovirales, family *Coronaviridae* and genus *Alphacoronavirus* (1). PEDV is enteropathogenic and causes diarrhea and vomiting, mainly in suckling pigs (2, 3). Although PEDV infects pigs of all ages, the severity of clinical signs and mortality is inversely related to the age of the pigs (4). PEDV primarily infects the epithelial lining of the intestinal mucosa, and the virus replicates in the cytoplasm of enterocytes of the small intestine (5). The progression of the infection causes villous atrophy and low numbers of infiltrating inflammatory cells into the affected areas, leading to malabsorptive diarrhea and dehydration (5–7). The early infection and innate immune responses towards PEDV at the intestinal epithelium are understudied, partly due to the lack of well-established, large-scale, and reproducible infection models alternative to traditional cell culture and animal models (8).

Virus isolation in cell culture is the primary method for identifying viable PEDV in different specimens (9–11). However, the commonly used cell lines (i.e., porcine epithelial/kidney cells or Vero cells) are convenient but may not fully replicate early immune interactions of PEDV infection. This is due to the lack of complex architecture of the intestinal mucosa that are presented in natural infection processes (12–15). Meanwhile, neonatal pig bioassays offer a more realistic model for studying viral infections and developing immunization strategies, diagnostics, and therapies (16, 17). Nevertheless, pig bioassays have limitations such as resource intensiveness, intrinsic variability from multiple donor animals, interference from the gut microbiome or secondary infections, and real-time host-virus interaction data collection challenges. Moreover, animal studies involving infectious agents must comply with “3R” (Replacement, Reduction, and Refinement) principles and the necessary biocontainment facilities.

Recent advancements in stem cell research have made significant progress towards establishing sufficiently robust and complex culture models to biologically and physiologically mimic the original tissue/organ of the animal. The development of 3D intestinal crypt cell-derived organoids (“enteroids” or “mini-guts”) (18) has emerged as a promising *in vitro* culture system to deepen our knowledge of the complex interactions between intestinal epithelial cells and enteric pathogens (19, 20). Organoids offer a strict *in vitro* culture environment more representative of cell complexity and microphysiology relevant to that *in vivo* than traditional cell culture (21).

Extracellular support matrix rich in laminin, collagen, and other extracellular matrix proteins, also known as “Matrigel”, are capable of differentiating porcine crypt stem cells into enteroids with complex cell phenotypes comprised of intestinal crypt cells, enterocytes, goblet cells, and Paneth cells (21–24). Although the Matrigel helps direct the polarity of the mucosal epithelium, it also complicates access to the apical surface or luminal side of the enteroids. Accessibility of the apical surface to viral particles, such as PEDV, is crucial for mimicking the natural cell-virus interactions *in vivo* (25).

Previous reports described the development of porcine enteroids (8, 23–27); however, the development characterization and comparison of enteroids derived from different segments of the small intestine within the context of infection were only briefly mentioned. The objectives of this study were to establish a porcine enteroids culture model with access to the luminal surface to study early infection and to assess the potential differences in the susceptibility in different segments of the porcine small intestines (i.e., duodenum, jejunum, and ileum).

## 2 Materials and methods

### 2.1 The study

**Figure 1:**
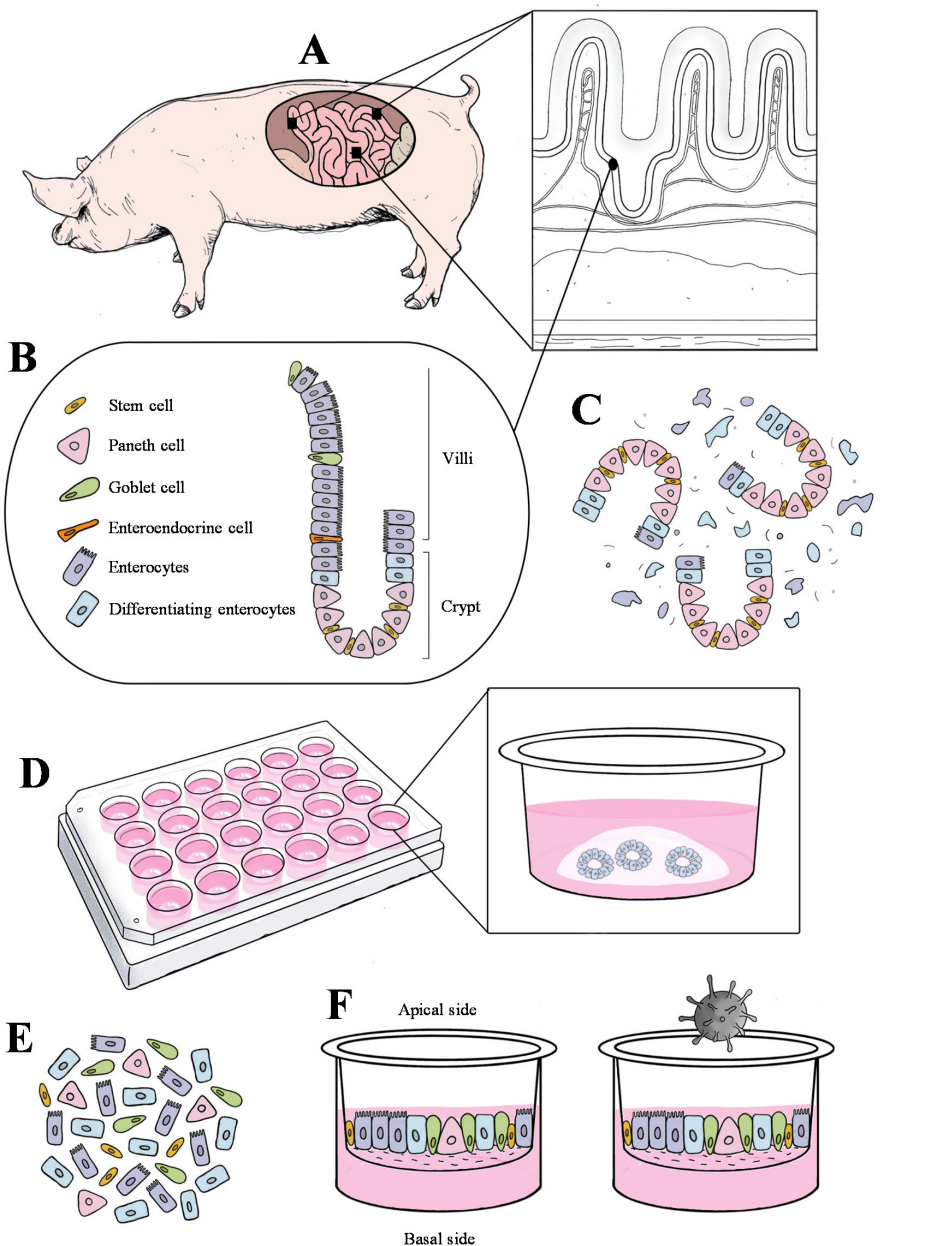
Schematic diagram of the crypt cell isolation, enteroids culture in Matrigel, and enteroids culture in transwell inserts. **(A)** Three segments of the small intestine (duodenum, jejunum, and ileum) were collected and processed from three cesarean-derived, colostrum-deprived (CD/CD) piglets. **(B)** Representative schema of multiple cell types in villus and crypt region from the pig intestinal mucosal epithelium. **(C)** Dissociation of crypt and villus region along with other cell debris. **(D)** Culturing crypt cells in Matrigel until they matured and differentiated into a complex 3D structure. **(E)** Dissociated enteroids. **(F)** Differentiated enteroids on the transwell with the apical side exposed, followed by virus inoculation.

A graphical overview of the study design is presented in **Figure 1**. Three major regions of the small intestine (duodenum/jejunum/ileum) were collected from four 7-10 days old cesarean-derived colostrum-deprived (CD/CD) pigs (inclusion criteria for this study). Crypt cells were isolated from the tissues and cultured in Matrigel for differentiation into 3D enteroids. The porcine 3D enteroids in Matrigel (PEMCs) were dissociated and subcultured on transwell inserts, allowing them to differentiate into polarized cells with exposed epithelial surfaces (PETCs). PETCs with apical brush border of enterocytes and other cell phenotypes derived from the duodenum, jejunum, and ileum were inoculated with PEDV, and the corresponding supernatants and cell pellets were collected to demonstrate PEDV infection.

### 2.2 Intestine collection from donor pigs

Donor CD/CD pigs (crossbreed between Large White and Landrace; Struve Labs, Manning, IA, USA) were euthanized simultaneously at 9 days of age following the animal care guidelines of the Iowa State University Institutional Animal Care and Use Committee (IACUC-20-028). Intestinal sections (3-5 cm) of the duodenum, jejunum, and ileum (i.e., three replicates per section, middle portion, processed together) were cut open longitudinally, and luminal contents were removed. The tissues were thoroughly washed with sterile phosphate-buffered saline, pH 7.4 (PBS; Thermo Fisher Scientific, Waltham, MA, USA) supplemented with 100 IU/mL of penicillin, 100 µg/mL of streptomycin (Thermo Fisher Scientific), 1.25 µg/mL of amphotericin-B (Thermo Fisher Scientific), 10 µg/mL amikacin (VWR International, Radnor, PA, USA), and 10 µg/mL ampicillin (VWR). After scrapping the mucus away using the blunt end of the scalpel (Integra, Mansfield, MA, USA), tissue sections were washed vigorously with PBS containing antibiotics and 2 mM N-acetyl cysteine (Millipore-Sigma, Burlington, MA, USA), and subsequently transported to the laboratory in ice-cold advanced Dulbecco’s Modified Eagle Medium: Nutrient Mixture F12 (Advanced DMEM/F-12; Thermo Fisher Scientific).

### 2.3 Isolation of porcine intestinal crypt cells

Tissue sections of the duodenum, jejunum, and ileum devoid of mucus were minced and collected into 15 mL conical tubes (Thermo Fisher Scientific) and pulse vortexed 8-10 times. The minced tissue was allowed to settle at the bottom of the tube and placed on ice for 5 min, and the supernatant was discarded. The tissue mince was serially washed five times in a chelating solution medium [5.6 mM sodium phosphate dibasic dihydrate (Na_2_HPO_4_-2H_2_O; Thermo Fisher Scientific), 7.9 mM potassium dihydrogen phosphate (KH_2_PO_4_; Thermo Fisher Scientific), 95.8 mM sodium chloride (NaCl; Millipore-Sigma); 1.6 mM potassium chloride (KCl; Millipore-Sigma), 0.35 mM sucrose (Millipore-Sigma), 54.89 mM D-Sorbitol (Thermo Fisher Scientific), 0.52 mM Dithiothreitol (DTT; Millipore-Sigma), 200 IU/mL of penicillin, 200 µg/mL of streptomycin, and 1.25 µg/mL of amphotericin-B]. Then, the tissue mince was incubated in a chelating solution medium containing 20 mM of ethylenediaminetetraacetic acid (EDTA; Thermo Fisher Scientific) at 4°C on a microplate shaker (VWR) at 100 rpm for 2 h to isolate crypt cells. The dissociation reaction with EDTA was neutralized using an equal volume of heat-inactivated EqualFetal bovine serum (eFBS; EqualFetal; Atlas Biologicals, Fort Collins, CO, USA) followed by cell straining through a 100 µm cell strainer (Greiner Bio-One, Monroe, NC, USA). The filtrate containing crypt cells was collected, washed in cold Advanced DMEM/F-12, and centrifuged at 100 x g for 5 min at 4°C to obtain the cell pellet, which was subsequently used for culturing porcine enteroids.

### 2.4 Harvesting of L-WRN medium

Following instructions from ATCC, L-WRN cells (CRL-3276; ATCC, Manassas, VA, USA) were cultured, and cell culture supernatants (“L-WRN conditioned medium”) were collected. L-WRN cells were derived from mouse fibroblast-like L-Wnt3A cells (ATCC CRL-2647™, ATCC) transfected with an R-spondin 3 and noggin co-expressing vector. The stable clones of L-WRN cells expressing Wnt3A, R-spondin 3, and noggin were selected using a medium containing G418 and Hygromycin B. In brief, L-WRN cells were first propagated with L-WRN culture medium (Advanced DMEM/F12 supplemented with 10% heat-inactivated eFBS, 100 IU/mL of penicillin, and 100 µg/mL of streptomycin), then selected with L-WRN culture medium and the addition of 0.5 mg/mL of Geneticin selective antibiotic (G418 Sulfate; Thermo Fisher Scientific), and 0.5 mg/mL of hygromycin B (Thermo Fisher Scientific). For subsequent passages, L-WRN cells were cultured in cell culture multi-flasks (Corning) with L-WRN culture medium only. After achieving 100% confluency, the cells were washed thoroughly with L-WRN culture medium exclusive of penicillin and streptomycin (“harvest medium”) and replaced with fresh harvest medium every 24 h after the collection of L-WRN conditioned medium. The collected L-WRN conditioned medium was centrifuged at 3,000 x g for 5 min to remove any cell debris, and stored in the same bottle at 4°C. This process was repeated for 12 consecutive days. After adding equal volumes of Advanced DMEM/F-12 to the collected L-WRN conditioned medium, the final medium was filtered through a 0.1 µm cell culture medium filter (Nalgene™, Thermo Fisher Scientific), aliquoted, and stored at -80°C until use.

### 2.5 Differentiation of porcine intestinal crypt cells in Matrigel

The porcine intestinal crypt cells isolated from each small intestine region were resuspended in ice-cold Matrigel [Matrigel growth factor reduced (GFR) basement membrane matrix, phenol red-free, LDEV-free; Corning, Tewksbury, MA, USA], dispensed into 24-well culture plates (Corning) as 30 µL droplets, and polymerized at 37°C for 10 min. Room temperature (20-22°C) enteroid medium [L-WRN medium supplemented with 100 µg/mL Primocin (*In vivo*Gen, San Diego, CA, USA), 5% heat-inactivated eFBS, 1X Glutamax (Thermo Fisher Scientific), 10 mM HEPES (Thermo Fisher Scientific), 1X B27 supplement (Thermo Fisher Scientific), 1X N2 supplement (Thermo Fisher Scientific), 1 mM N-acetyl cysteine (Millipore-Sigma), 50 ng/mL epidermal growth factor (EGF; PeproTech, Cranbury, NJ, USA), 10 nM Leu15-gastrin I human (Millipore-Sigma), 10 mM nicotinamide (Millipore-Sigma), 0.5 µM transforming growth factor beta inhibitor A83-01 (TGFβ; Tocris, R&D Systems, Minneapolis, MN, USA), and 10 µM p38 MAPK inhibitor SB202190 (Millipore-Sigma)], was added to each well of the cell culture plate (500 µL) containing the polymerized Matrigel droplet with crypt cells.

Following 48 h post-propagation, enteroid medium supplemented with 10 µM Rho kinase inhibitor Y-27632 (ROCKi; Reprocell, Beltsville, MD, USA) and 2.5 µM Glycogen synthase kinase 3 b inhibitor CHIR99021 (GSKi; Reprocell) was used. After 5-7 days, the porcine intestinal crypt cells differentiated into PEMCs, with their corresponding lumen, crypt, and villus region. For subculturing, PEMCs were collected in ice-cold advanced DMEM/F12 from each well and centrifuged at 100 x g for 5 min at 4°C. After discarding the supernatant, the cell pellets were treated with 1X TrypLE express without phenol red (Thermo Fisher Scientific) for 6 min at 37°C. TrypLE express dissociation was neutralized with an equal volume of heat-inactivated eFBS. The dissociated cells were subcultured in Matrigel or in transwell inserts [0.4 µm pore size; polyethylene terephthalate (PET) membrane; 2 x 10^6^ cm^2^ pore density; Greiner Bio-One] or frozen in a freezing medium containing 10% dimethyl sulfoxide (DMSO; Millipore-Sigma), 30% advanced DMEM/F-12, 60% heat-inactivated eFBS for future use. For cellular characterization, PEMCs were either fixed with 4% paraformaldehyde (Electron Microscopy Sciences, Hatfield, PA, USA) for 15 min for immunohistochemistry (IHC) staining, or collected in cold TRIzol reagent (Thermo Fisher Scientific), placed for 10 min at room temperature for RNA isolation.

### 2.6 Culture of polarized porcine enteroids on culture inserts or transwells

The dissociated PEMCs derived from the duodenum, jejunum, and ileum were seeded on transparent 24-well transwell inserts precoated with advanced DMEM/F12 containing 1% Matrigel and 10 µg/mL collagen from the human placenta-Bornstein and Traub Type IV (Millipore-Sigma). A cell density of ~5,000 cells/mm^2^ per well was inoculated on each transwell insert using the enteroids medium supplemented with ROCKi and GSKi. The platewell was inoculated with 500 µL of the medium mentioned above. The enteroids medium was replaced every 48 h to allow PEMCs to grow and differentiate into PETCs for up to a week. Cells were either fixed in 4% paraformaldehyde (Electron Microscopy Sciences) for 15 min or lysed in cold TRIzol reagent placed for 10 min at room temperature for cellular and immunological characterization.

### 2.7 Cell proliferation of PEMCs and PETCs

A CellTiter-Glo 3D Cell Viability Assay (Promega, Madison, WI, USA) was used, according to the manufacturer’s instructions, to characterize the growth kinetics of region-specific (duodenum, jejunum, and ileum) PETCs. In brief, the CellTiter-Glo 3D reagent was added in a 1:1 proportion to the enteroids medium and incubated for 30 min for cell lysis. The lysate was transferred to a white lumitrac 96-well microplate (Greiner Bio-One), with the luminescence detected through an EnVision XCite 2105 Multimode Plate Reader (Revvity, Waltham, MA, USA). The level of detected luminescence corresponds to the adenosine triphosphate (ATP) generated from the PETCs, which is directly proportional to cell viability. The data was presented as Relative luminescence units (RLU), normalized via the subtraction of the mean luminescence parameter of the blank controls (medium-only) from the luminescence parameters of the PETCs.

### 2.8 Cellular characterization of porcine enteroids

#### 2.8.1 Tissue, PEMCs, and PETCs processing for staining

Staining of all porcine intestinal tissues, PEMCs, and PETCs was performed using paraffin-embedded and cross-sectioned blocks. After collection, processing, and transport to the laboratory, the small intestine tissues from pigs were fixed in 10% buffered formalin (Millipore-Sigma) at room temperature for 24 h, and processed for routine histopathologic examination. In comparison, PEMCs and PETCs were fixed in 4% paraformaldehyde, as described in previous sections. To overcome the low yield of the fixed PEMCs in paraffin blocks due to the difficulty of holding enteroids during the routine procedure of dehydration and paraffin embedding, PEMCs were resuspended in agarose plugs. Once fixed, PEMCs were collected, washed twice with ice-cold PBS pH 7.4, and centrifuged at 300 x g for 5 min at 4°C to remove the Matrigel. The enteroids were resuspended in 2% Ultrapure Agarose (Thermo Fisher Scientific) and transferred to a cut-open 1000 μL tip mold on an ice block. Once the agarose suspension solidified, the agarose plugs were stored in 70% ethanol (VWR) until the routine processing of dehydration and paraffin embedding. As for the fixed PETCs, the transwell membranes were slit from the opposite side, covered with TissueWrap biopsy papers (CellPath, StatLab Medical Products, McKinney, TX, USA), and stored in 70% ethanol at room temperature until the routine processing of dehydration and paraffin embedding was performed (28, 29). The paraffin-embedded tissues, PEMCs, and PETCs were sectioned into 4 μm cross sections and mounted on positive-charged glass slides (Thermo Fisher Scientific) for subsequent staining.

The paraffin-embedded tissue and cell blocks were deparaffinized and stained with hematoxylin and eosin (H&E) using the Tissue-Tek Prisma Plus Automated Slide Stainer (Sakura Finetek USA Inc., Torrance, CA, USA) (30). Likewise, neutral and acidic mucins in porcine tissue, PEMCs, and PETCs were detected through periodic acid-Schiff (PAS) and alcian blue staining, respectively (30). After counterstaining, the sections were cleared in Histo-Clear II (Electron Microscopy Sciences) and mounted in Tissue-Tek Glas mounting medium (Sakura Finetek USA, Inc).

#### 2.8.2 Immunohistochemistry and immunocytochemistry

Deparaffinized porcine tissue, PEMCs, and PETCs slides were treated with 11.6 mM sodium citrate buffer (Millipore-Sigma), pH 6.0 at 96°C for 30 min (porcine tissue and PEMCs) and 10 min (PETCs). Slides were washed three times with tris-buffered saline pH 7.6 (TBS; Millipore-Sigma) with 0.1% Tween 20 (TBST; Millipore-Sigma) and incubated with Animal-Free Blocker^®^ R.T.U (Vector Laboratories, Newark, CA, USA) for 30 min at room temperature. Sections were then incubated with the respective primary antibodies using the appropriate dilutions with Animal-Free Blocker^®^ R.T.U (**Table 1**) overnight (16 h) at 4°C. The PEMCs and PETCs slides were washed twice with TBST and applied with 1% hydrogen peroxide (Sam’s Club, Bentonville, AR, USA) for 1 h and 10 min. After washing three times with TBST, sections were incubated with the undiluted secondary antibody VisUCyte™ HRP Polymer, as per manufacturer instructions (R&D System, Minneapolis, MN, USA) for 30 min at room temperature and treated with ImmPACT^®^ diaminobenzidine (DAB) EqV Substrate (Vector Laboratories) for 5 min. Lastly, the slides were rinsed with distilled water, counterstained with hematoxylin for approximately 10 s, mounted with Tissue-Tek Glas Mounting Medium, and covered with a cover glass (Leica Biosystems, Nußloch, Germany), and observed under an inverted fluorescent microscope (Olympus CKX41; Olympus Corporation, Waltham, MA, USA) equipped with an Infinity 2 camera operated with Infinity analyze v6.5.5 software (Teledyne Lumenera, Ottawa, ON, Canada).

**Table 1:** Summary of the antibodies used in the immunohistochemistry and immunocytochemistry in porcine tissues, porcine enteroids Matrigel culture (PEMCs) and porcine enteroids transwell culture (PETCs) in this study.

Company	Antibody	Catalog number	Clone	Clonality	Raised	Concentration
Santa Cruz Biotechnology	Pan cytokeratin	sc-81714	AE1/AE3	IgG1	Mouse monoclonal	0.2 µg/ml
Bio-Rad	Pan cytokeratin	MCA1907T	AE1/AE3 Cocktail	IgG1	Mouse monoclonal	0.2 µg/ml
Santa Cruz Biotechnology	Villin 1	sc-58897	1D2C3	IgG1	Mouse monoclonal	4 µg/ml
Novus Biologicals	Chromogranin A	NBP2-32956	LK2H10	IgG1	Mouse monoclonal	0.2 µg/ml
Santa Cruz Biotechnology	Lysozyme C	sc-518012	E-5	IgG1	Mouse monoclonal	4 µg/ml
Santa Cruz Biotechnology	PCNA	sc-56	PC10	IgG2	Mouse monoclonal	0.8 µg/ml
Novus Biologicals	LGR5	NBP1-28904SS	–	–	Rabbit polyclonal	2 µg/ml
Thermo Fisher Scientific	ZO-1	33-9100	ZO1-1A12	IgG1	Mouse monoclonal	10 µg/ml
Thermo Fisher Scientific	E-Cadherin	33-4000	4A2C7	IgG1	Mouse monoclonal	2.5 µg/ml
Thermo Fisher Scientific	Occludin	71-1500	–	–	Rabbit polyclonal	1 µg/ml

#### 2.8.3 RNA isolation, reverse transcription, and qPCR

Total RNA was extracted from tissues, PEMCs, and PETCs using a TRIzol reagent and chloroform phase separation, followed by column-based extraction using a commercially available kit (QIAGEN, Hilden, Germany). For intestinal tissues, snap-frozen tissue samples were processed by bead-beating using 2.8 mm ceramic beads (Omni International, Kennesaw, GA, USA). Total RNA concentration was measured using a NanoDrop one microvolume UV-Vis spectrophotometer (Thermo Fisher Scientific). Reverse transcription was performed in samples with A260/280 between 1.96 and 2.05 using the qScript XLT cDNA SuperMix Kit (Quantabio, Beverly, MA, USA) with 150 ng of total RNA.

All quantitative polymerase chain reactions (qPCR) were conducted using 1X PowerUp SYBR Green Master Mix (Applied Biosystems, Thermo Fisher Scientific), 500 nM of swine-specific primers (**Table 2**), 1.5 ng (cells), and 20 ng (tissues) of total RNA converted to complementary DNA (cDNA). The reactions were performed on an ABI 7500 Fast Real-Time PCR thermocycler (Thermo Fisher Scientific) under the following cycling conditions: 50°C for 2 min and 95°C for 2 min holding; 40 cycles, 95°C for 15 s denaturation and 60°C for 1 min amplification. The final melting curve analysis was performed at 95°C for 15 s, 60°C for 1 min, and 95°C for 15 s. All qPCR reactions were run in duplicate, and no-template controls (NTC) were included in each plate. Data analysis was carried out using 7500 Software v2.3 (Thermo Fisher Scientific), and the results were exported to Microsoft Excel (Microsoft Corporation, Redmond, WA, USA). Samples showing multiple melting peaks crossing the 36 cycle threshold (Ct) were excluded from the analysis.

**Table 2:** Summary of various cell marker expressions (immunohistochemistry/immunocytochemistry and gene expression) in the tissues, PEMCs and PETCs derived from 3 regions (duodenum, jejunum, and ileum) of the porcine small intestine.

			Tissue segments			PEMCs^a^			PETCs^b^		
Cell-specific markers		Cell specificity	D^c^	J^d^	I^e^	D	J	I	D	J	I
IHC/ICC^f^ expression Catalog number											
Pan cytokeratin	sc-81714	Epithelial cells	+	+	+	+	+	+	+	+	+
Villin 1	sc-58897	Enterocytes	+	+	+	+	+	+	+	+	+
Mucin	–	Goblet cells	+	+	+	+	+	+	+	+	+
Chromogranin A	NBP2-32956	Enteroendocrine cells	+	+	+	+	+	+	+	+	+
Lysozyme C	sc-518012	Paneth cells	+	+	+	+	+	+	+	+	+
PCNA	sc-56	Proliferation	+	+	+	+	+	+	+	+	+
LGR5	NBP1-28904SS	Stem cell	+	+	+	+	+	+	+	+	+
ZO-1	33-9100	Tight Junction Protein	+	+	+	+	+	+	+	+	+
E-Cadherin	33-4000	Adherens Junction Protein	+	+	+	+	+	+	+	+	+
Occludin	71-1500	Tight Junction Protein	+	+	+	+	+	+	+	+	+
Gene expression NCBI accession number and primer sequence reference											
*PCNA*	NM_001291925.1 (31)	Proliferation	+	+	+	+	+	+	+	+	+
*MUC2*	XM_021082584.1 (22)	Goblet cells	+	+	+	+	+	+	+	+	+
*CHGA*	NM_001164005.2 (22)	Enteroendocrine	+	+	+	+	+	+	+	+	+
*SLC5A1/SGLT1*	NM_001164021.1 (22)	Mature enterocytes	+	+	+	+	+	+	+	+	+
*LYZ*	NM_214392.2 (22)	Paneth cells	+	+	+	+	+	+	+	+	+
*FZD5*	XM_005672133.3 (32)	Paneth cells	+	+	+	+	+	+	+	+	+
*EPHB2*	XM_005656047.3 (32)	Paneth cells	+	+	+	+	+	+	+	+	+
*LGR5*	XM_021090898.1 ^*^	Crypt cells	+	+	+	+	+	+	+	+	+

^a^ Porcine enteroids in Matrigel culture; ^b^ Porcine enteroids on transwell culture; ^c^ Duodenum; ^d^ Jejnum; ^e^ Ileum; ^f^ Immunohistochemistry/Immunocytochemistry. Proliferating cell nuclear antigen (PCNA); Mucin 2 (MUC2); Chromogranin A (CHGA); Solute carrier family 5 member 1 (SLC5A1/SGLT1); Lysozyme (LYZ); Frizzled class receptor 5 (FZD5); Ephrin type-B receptor 2 (EPHB2); Leucine-rich repeat-containing G protein-coupled receptor 5 (LGR5). LGR5 Forward primer (5’-3’) GGTCGGTTGCCAAATCGTT; Reverse primer (3’-5’) TCCAGGGCTGCCAGAGTAAG. * The sequences of the forward primer (5’-3’) GGTCGGTTGCCAAATCGTT and reverse primer (3’-5’) TCCAGGGCTGCCAGAGTAAG were designed for this study.

### 2.9 PEDV propagation and titration

Vero 81 cells (CCL-81; ATCC) were used to propagate the PEDV non-S-INDEL strain USA/Colorado/2013 as described previously (8). Briefly, Vero 81 cells were seeded in a 175 cm^2^ tissue culture flask (Nunc, Thermo Fisher Scientific) using growth medium [– High Glucose (Millipore-Sigma) supplemented with 10% heat-inactivated FBS (ATCC), 100 IU/mL of penicillin, 100 µg/mL of streptomycin, and 25 μg/mL of gentamicin (Thermo Fisher Scientific)] and incubated in a humidified incubator at 37°C with 5% CO_2_ for 24 h. Cell monolayers with 80% confluence were used for virus propagation, infection medium (growth medium without heat-inactivated FBS) supplemented with 6 µg/mL trypsin (Millipore-Sigma) was used for mock- or PEDV-inoculation of the cells. After 1 h incubation at 37°C with 5% CO_2,_ the inoculum was removed, and cells were washed twice with PBS pH 7.4 and replaced with fresh infection medium. The inoculated cells were incubated until noticeable cytopathic effects (CPE) were observed in the cell monolayer, approximately 48 h post-inoculation (hpi). The virus was harvested by performing three freeze/thaw cycles, followed by centrifugation at 3,000 x g for 20 min at 4°C to remove the cellular debris, aliquoted, and preserved in liquid nitrogen for further use. The 50% tissue culture infective dose (TCID_50_) per mL of the virus stock was obtained using the Spearman and Karber method (33, 34).

### 2.10 PEDV inoculation in PETCs

The susceptibility of region-specific PETCs to PEDV infection was determined using fully differentiated PETCs (5 days after seeding on transwells) derived from the duodenum, jejunum, and ileum. PETCs were washed with advanced DMEM/F-12 (Thermo Fisher Scientific) and preincubated with transwell infection medium [Advanced DMEM/F12 supplemented with 100 µg/mL Primocin, 1X B27 supplement, 1X N2 supplement, 2% Ultroser G (Sartorius, Göttingen, Germany), 1X Glutamax, 10 mM HEPES; 1 mM N-acetylcysteine; 10 nM gastrin] 24 h before PEDV inoculation. Subsequently, PETCs were either inoculated with PEDV (1.58 x 10^5^ TCID_50_/mL) or mock-inoculated with transwell infection medium supplemented with 2 µg/mL of 0.1% trypsin (Millipore-Sigma) and incubated for 2 h at 37°C, with 5% CO_2_. After 2 h, the inoculum was removed, and both the transwell inserts and platewells were thoroughly washed with advanced DMEM/F-12 and replaced with fresh 300 µL transwell infection medium. The inoculated PETCs were further incubated for 22 h at 37°C with 5% CO_2_. To end the infection, the cells were fixed with 80% acetone (Thermo Fisher Scientific) for immunofluorescence assay (IFA), or lysed with cold TRizol reagent for PEDV RNA isolation.

#### 2.10.1 PEDV IFA and image analysis

PETCs were fixed with 80% ice-cold acetone for 15 min and air-dried for 30 min at room temperature. After washing twice each well with 200 µL PBS, 100 µL of fluorescein isothiocyanate (FITC)-conjugated mouse anti-PEDV nucleocapsid (N) protein IgG1 monoclonal antibody (Medgene, Brookings, SD, USA) diluted 1:100 in PBS pH 7.4 containing 0.1% bovine serum albumin (BSA; Jackson Immuno Research, West Grove, PA, USA) was added per well and incubated for 1 h at 37°C. After incubation, PETCs were washed twice with PBS pH 7.4, and imaged using a CKX41 inverted microscope (Olympus) equipped with an INFINITY 2 camera. Images were captured at 200 X magnification using Infinity 2 using Infinity Analyze software v6.5.5 (Teledyne Lumenera). The color channels on an image were split using ImageJ v1.54f software (35), and the green channel was selected for quantitative analysis. To reduce non-specific background noise the “Subtract Background” function with a “Rolling ball radius” of 150 was applied. The area containing specific fluorescent signals was defined by setting an intensity threshold from 30-225, and the fluorescent signals within this area were quantified. Mean Fluorescence Intensity (MFI), percentage of area with fluorescence signal, and Integrated Fluorescence Intensity (product of MFI and area) were calculated for each image using ImageJ software. Integrated Fluorescence Intensity was used as parameter to compare fluorescence signal intensity.

#### 2.10.2 PEDV RNA extraction and RT-qPCR

PEDV RNA detection was performed in both the supernatants and PETCs. Cold TRIzol reagent was used to lyse and inactivate PEDV after 10 min of incubation at room temperature. Following phase separation with chloroform (Fisher Scientific), a viral RNA extraction kit (Omega Bio-tek, Norcross, GA, USA) was used to extract PEDV RNA. The isolated RNA was then used for quantitative reverse transcription-PCR (RT-qPCR) assay. The RT-qPCR reactions (20 µL) included 2X Power SYBR Green RT-PCR Master Mix with 125X RT Enzyme Mix (Applied Biosystems, Thermo Fisher Scientific), 800 nM forward and reverse primers for the PEDV *N-gene* (36), DNase/RNase-Free ultra-pure distilled water (Thermo Fisher Scientific), and 3 µL of the extracted RNA sample. All samples were tested in duplicate, with a “no template” control (NTC) included in each run. The RT-qPCR assays were run on an ABI 7500 Fast Real-Time PCR thermocycler (Thermo Fisher Scientific) with cycling conditions, 48°C for 30 min and 95°C for 10 min holding; 40 cycles, 95°C for 15 s denaturation and 60°C for 1 min annealing. A melting curve was included, with 1 cycle of 95°C for 15 s, 60°C for 1 min, and 95°C for 15 s. The RT-qPCR results were analyzed using 7500 Fast System Software (Thermo Fisher Scientific), and samples with a Ct value above 36 were considered negative.

For normalization, six endogenous control genes (*ACTB, B2M, EIF3K, GAPDH, PPIA, RPS9*) were analyzed using qBase+ gene expression analysis software (Bio-gazelle, Zwijnaarde, Belgium). The software calculates the stability of endogenous control genes and provides an M-value, with *EIF3K* identified as the most stable endogenous control gene for relative quantitation based on its low M-value (37) in both PEDV- and mock-inoculated cultures. PEDV *N-gene* was normalized using the *EIF3K* gene, and ΔΔCt relative quantitation (RQ) analysis was conducted as described previously (38).

### 2.11 Statistical analysis

The relative luminescence data were generated from one biological replicate with two technical replicates for cell proliferation assay. For PEDV infectious studies in PETCs, data were obtained from four pigs (biological replicates). Integrated fluorescent intensity data were obtained from image analysis of two 200X magnified images from each group. Statistical analysis was performed using GraphPad Prism 10.1.2 (GraphPad Software, San Diego, CA, USA). A two-way analysis of variance (ANOVA) with multiple comparisons and Tukey’s correction was conducted to determine significant differences in the tested groups. Analysis was performed on integrated fluorescence intensity and average RQ (PEDV *N-gene/EIF3K*) values between PEDV- and mock-inoculated PETCs groups derived from different intestinal segments. Data are presented as mean ± standard error of the mean (SEM), with significance defined as a *P* value of < 0.05.

## 3 Results

### 3.1 PEMCs and PETCs established from the duodenum, jejunum, and ileum showed no significant morphological differences

**Figure 2:**
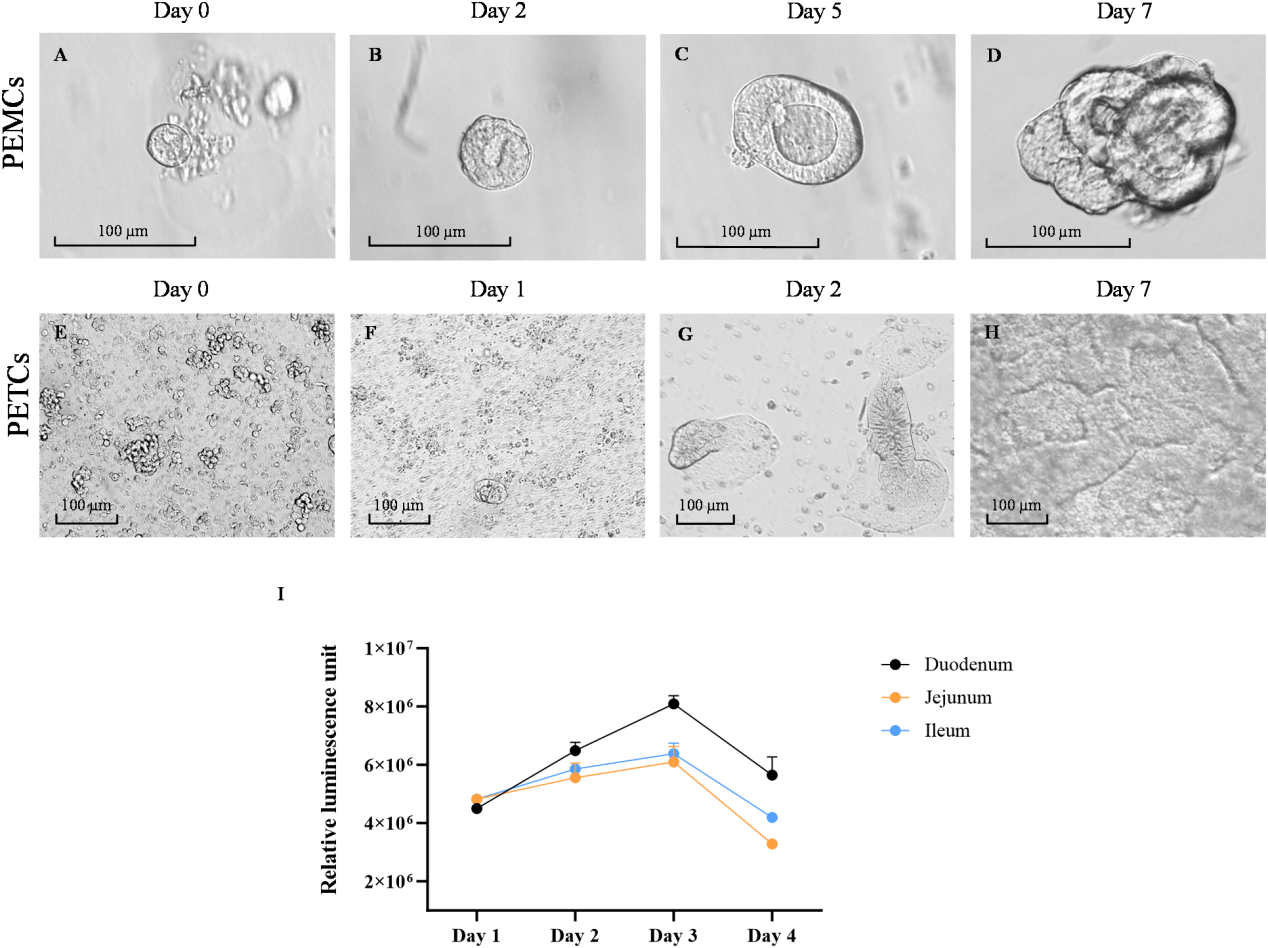
Growth of porcine enteroids on matrigel and transwell cultures. Representative images of growth progression porcine enteroids in Matrigel culture (PEMCs) **(A–D)** and porcine enteroids in transwell culture (PETCs) **(E–H)** at various stages. **(A)** Proliferation of porcine crypt cells in Matrigel, started budding with spheroid-like morphology and less than 100 µm. **(B)** Porcine crypt cells were enlarged in size with lumen formation by day 2. **(C)** Porcine crypt cells continued to expand in size with evident lumens in the center and protrusion of multilobular morphology starting to emerge by day 5. **(D)** Porcine crypt cells differentiated into a more complex multilobular morphology, defining it as enteroid. **(E)** Dissociated enteroids were observed as single-rounded cells when first seeded onto the transwell inserts coated with 10 µg/mL collagen and 1% Matrigel. **(F)** Multiple small islands of cells in the first few days after seeding. **(G)** The small island of cells expanded into a monolayer of cells on the transwell insert. **(H)** Expanding multiple islands of cells fuse to form a confluent monolayer on top of the transwell inserts by day 7, defining as enteroids on the transwell. Scale bar = 100 µm. Line graph showing cell growth kinetics of the PETCs derived from the three small intestine segments using CellTiter-Glo 3D Cell Viability Assay (Promega) **(I)**. Relative luminescence units (RLU) on the y-axis were plotted against time on the x-axis. This graph was generated using the average of two technical replicates of one biological replicate (1 pig); Error bars = SEM.

The yield of crypt cells isolated varied across the three regions of the pig’s small intestine, as evidenced by differences in cell pellet size following tissue dissociation. The ileum and jejunum yielded larger cell pellets, while crypt cells from the duodenum appeared cleaner with less tissue debris. During the first 24 to 48 h of culture, crypt cells underwent differentiation into an aggregate cell morphology, also known as spheroid-like structures (**Figure 2A**), and expanded in size (**Figure 2B**). These spheroid-like cells began budding and differentiating, displaying typical multilobular morphology by day 5 (**Figure 2C**). By day 7, crypt cells had fully differentiated into 3D PEMCs, exhibiting villus, crypt, and luminal domains (**Figure 2D**).

Trypsin-dissociated PEMCs derived from the three regions of the small intestine were initially seeded on transwell inserts, forming multiple islands of cells attached to the membrane (**Figures 2E, F**). By day 2, these cells expanded into isolated budding bodies (**Figure 2G**), later fusing to form a uniform monolayer. The cells demonstrated active replication, with enterocytes exhibiting brush border-like formations (**Figure 2G**). By day 7, a confluent monolayer with an accessible luminal side facing the top of the transwell insert defined the formation of PETCs (**Figure 2H**).

Using the CellTiter-Glo 3D Cell Viability Assay, the viability and growth rate of PETCs derived from the three different small intestinal segments were evaluated (**Figure 2I**). The RLU increased from day 1 to 3 and decreased by day 4 regardless of the segment from which PETCs were derived. Although duodenum-derived PETCs showed a trend of higher luminescence signal parameters, no statistically significant differences were observed in growth kinetics among PETCs derived from the three segments of the small intestine.

**Figure 3:**
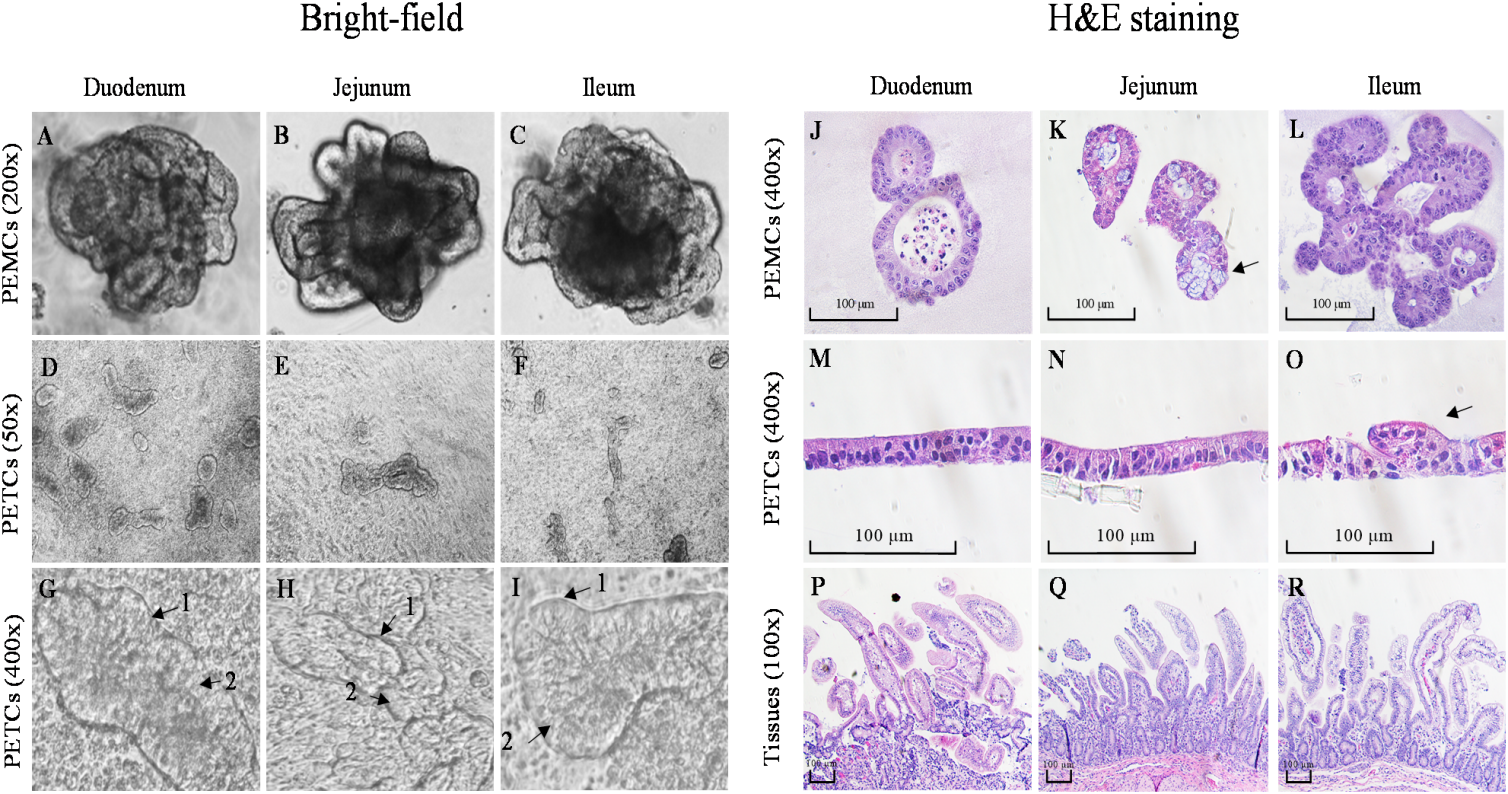
Comparative morphology of bright-field and H&E staining images of porcine enteroids Matrigel culture (PEMCs), porcine enteroids transwell culture (PETCs), and intestinal tissue from 3 regions (duodenum, jejunum, and ileum) of porcine small intestine. **(A–C)** Bright-field images of 5 to 7-day-old differentiated enteroids in Matrigel with crypt and villus-like domains at 200X magnification (cropped images). **(D–F)** Bright-field images of 5-day-old enteroids grown on transwell insert at 50X magnification. **(G–I)** Enteroids grown on transwell insert at 400X magnification (cropped images); note the brush border (1) and enterocytes (2). **(J–L)** H&E staining of paraffin-embedded 5-7 day-old PEMCs cross segments at 400X magnification; note the presence of goblet cells in K (arrow). **(M–O)** H&E staining of paraffin-embedded 5-7 day-old PETCs across segments at 400X magnification, note the morphology of the protruding villi in O (arrow); **(P–R)** H&E staining of paraffin-embedded porcine small intestinal tissue cross-segments at 100X magnification.

In contrast, PEMCs from the ileum and jejunum exhibited comparatively faster growth during early passages based on morphological observations. Albeit no major differences were noted in subsequent passages across regions for both PEMCs (**Figures 3A–C**) and PETCs (**Figures 3D–F**). PETCs displayed villus-like domains lying flat on transwell inserts with clear demarcations between cells and brush border-like domains (**Figures 3G–I**).

H&E stained PEMCs (**Figures 3J–L**) revealed enterocytes with a brush border oriented towards the lumen, containing sloughed cell and mucus, demonstrating regional specialization with both crypt and villus regions, with Matrigel surrounding the cells. In PETCs (**Figures 3M–O**), the enterocyte brush border was exposed towards the apical surface, and drooping villi-like structures (**Figure 3O**) were observed with cells attaching to the transwell membrane. The polarity of cell growth for PEMCs and PETCs was demonstrated in comparison with the porcine small intestinal tracts (**Figures 3P–R**). However, no apparent differences in cell morphology were observed between PEMCs and PETCs across the three regions.

### 3.2 Cell phenotyping of PEMCs and PETCs derived from different segments of the small intestine resembled the porcine intestinal epithelium

**Figure 4:**
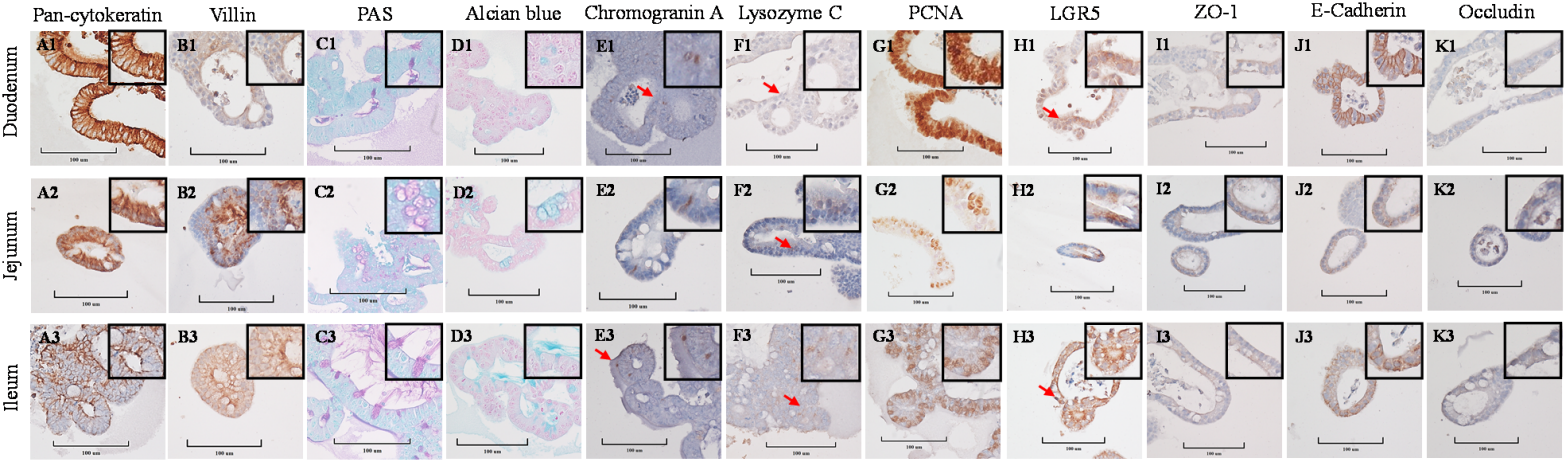
Cellular characterization of paraffin-embedded day 5-7 porcine enteroids in Matrigel culture (PEMCs) cross segments derived from three porcine small intestine regions (duodenum, jejunum, ileum) at 400X magnification (cropped images). **(A1–A3)** Epithelial cell marker pan-cytokeratin. **(B1–B3)** Brush border of the enterocyte marker villin. **(C1–C3)** Periodic acid-Schiff (PAS) stain showing magenta red for neutral mucins. **(D1–D3)** Alcian blue stain demonstrating acidic mucins. **(E1–E3)** Neuroendocrine cell marker Chromogranin A. (F1–F3) Paneth cell marker lysozyme C. **(G1–G3)** Cell proliferation marker PCNA. **(H1–H3)** Stem cell marker LGR5. **(I1** to **I3)** Tight-junction marker ZO-1. **(J1–J3)** Adheren-junction marker E-Cadherin. **(K1–K3)** Tight-junction marker Occludin.

**Figure 5:**
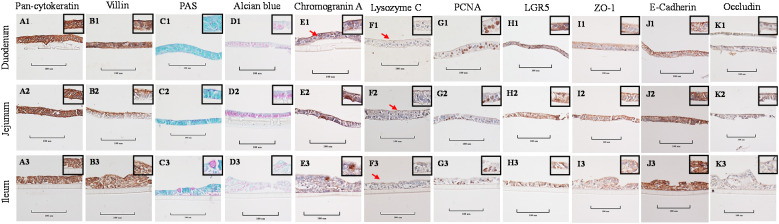
Cellular characterization of paraffin-embedded day 5-7 porcine enteroids on transwell culture (PETCs) cross segments derived from three porcine small intestine regions (duodenum, jejunum, ileum) at 400X magnification (cropped images). **(A1–A3)** Epithelial cell marker pan-cytokeratin. **(B1–B3)** Brush border of the enterocyte marker villin. **(C1–C3)** Periodic acid-Schiff (PAS) stain showing magenta red for neutral mucins. **(D1–D3)** Alcian blue stain demonstrating acidic mucins. **(E1–E3)** Neuroendocrine cell marker Chromogranin A. **(F1–F3)** Paneth cell marker lysozyme C. **(G1–G3)** Cell proliferation marker PCNA. **(H1–H3)** Stem cell marker LGR5. **(I1–I3)** Tight-junction marker ZO-1. **(J1–J3)** Adheren-junction marker E-Cadherin. **(K1–K3)** Tight-junction marker Occludin.

**Figure 6:**
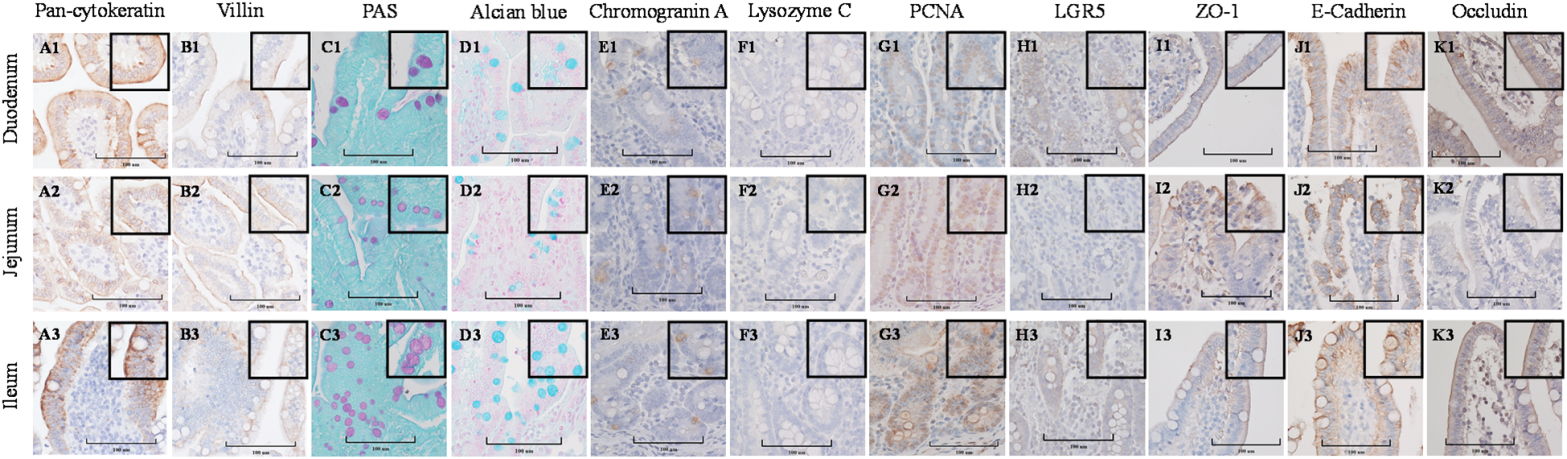
Cellular characterization of paraffin-embedded tissue cross segments derived from three porcine small intestine regions (duodenum, jejunum, ileum) of 7-10 day-old cesarean-derived, colostrum-deprived (CD/CD) neonatal piglets at 400X magnification (cropped images). **(A1–A3)** Epithelial cell marker pan-cytokeratin. **(B1–B3)** Brush border of the enterocyte marker villin. **(C1–C3)** Periodic acid-Schiff (PAS) stain showing magenta red for neutral mucins. **(D1–D3)** Alcian blue stain demonstrating acidic mucins. **(E1–E3)** Neuroendocrine cell marker Chromogranin A. **(F1–F3)** Paneth cell marker lysozyme C. **(G1–G3)** Cell proliferation marker PCNA. **(H1–H3)** Stem cell marker LGR5. **(I1–I3)** Tight-junction marker ZO-1. **(J1–J3)** Adheren-junction marker E-Cadherin. **(K1–K3)** Tight-junction marker Occludin.

Stained cross-sections of PEMCs (**Figure 4**) and PETCs (**Figure 5**) derived from the duodenum, jejunum, and ileum were positive by immunocytochemistry (ICC) for markers representing multiple cell types representatives of the porcine intestinal epithelia *in vivo* (**Figure 6**). These markers included epithelial cell (Pan-cytokeratin); brush border of enterocyte (Villin-1); neutral (PAS) and acidic (Alcian blue) mucin secretion of goblet cells; enteroendocrine cell (chromogranin A); Paneth cell (Lysozyme); proliferating cell nuclear antigen (PCNA) marker, leucine-rich repeat containing G protein-coupled receptor (LGR5) stem cell marker, and epithelial barrier markers (ZO-1, E-Cadherin, and Occludin). In addition, gene expression analysis across porcine tissues, PEMCs, and PETCs from different segments of the small intestine further supported these findings. The analysis revealed expression of genes associated to various cell types, i.e., stem cells (*LGR5*), enterocytes (*SLC5A1/SGLT1*), goblet cells (*MUC2*), Paneth cells (*LYZ, FZD5, and EPHB2*), enteroendocrine cells (*CHGA*), and proliferation marker (*PCNA*) (**Table 2**).

### 3.3 PETCs established from different segments of the small intestine were susceptible to PEDV infection, particularly jejunum-derived PETCs

**Figure 7:**
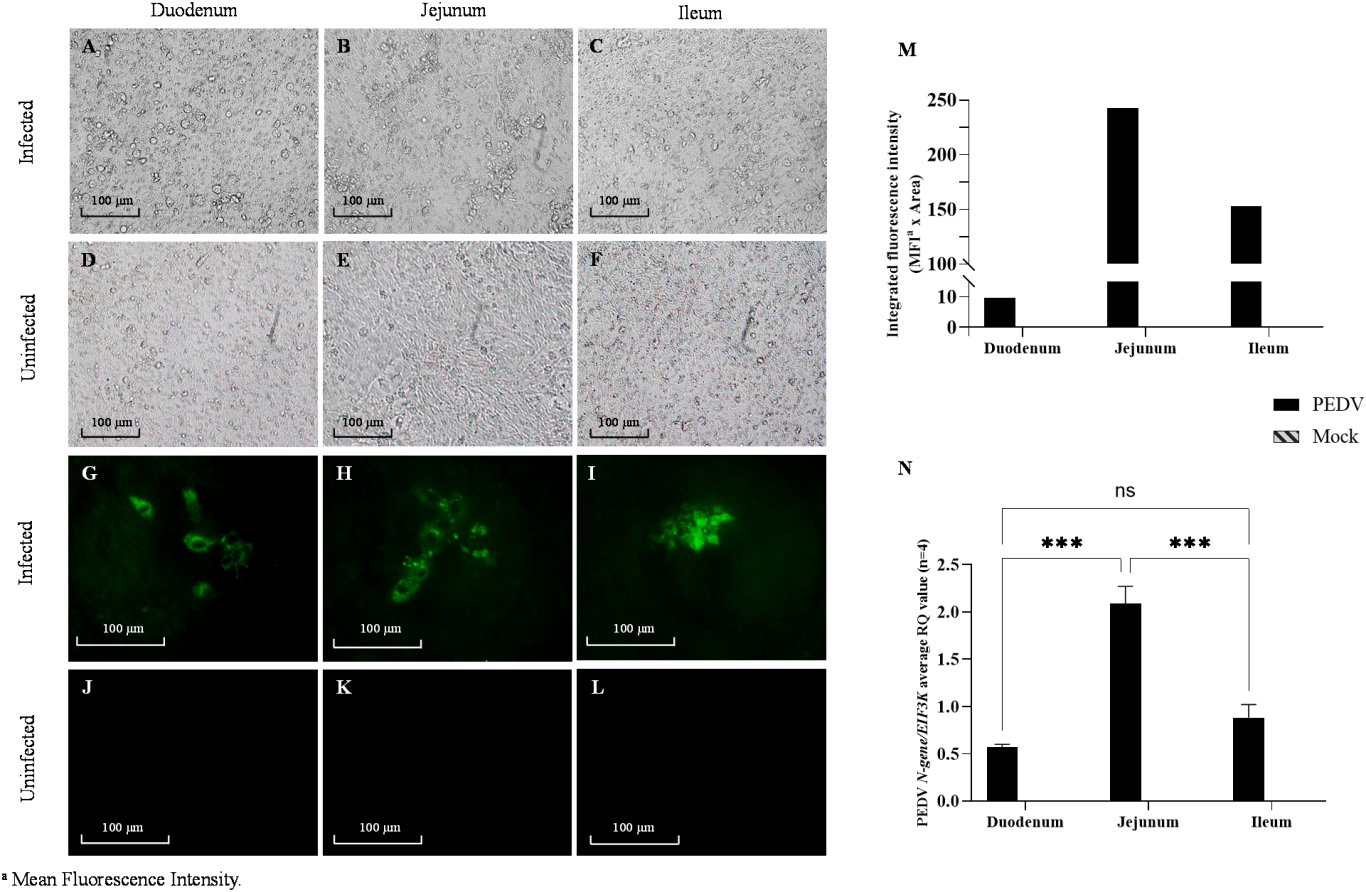
Porcine enteroids on transwell culture (PETCs) derived from three regions (duodenum, jejunum, and ileum) of porcine small intestine inoculated with PEDV and mock. Representative images of PETCs inoculated with porcine epidemic diarrhea virus (PEDV, USA/Colorado/2013) at 1.58 x 10^5^ TCID50/mL or mock-inoculated and incubated for 24 h (n=4); Bright-field images of PEDV-inoculated cells showing cytopathic changes such as increased detachment of cells **(A–C)** as compared to the mock-inoculated **(D–F)**. Immunofluorescence images showing for PEDV nucleocapsid (N) protein (green) following staining with an IgG1 fluorescein isothiocyanate (FITC)-conjugated anti-PEDV N protein monoclonal antibody (Medgene) at 1:100 dilution in PBS pH 7.4 with 0.1% bovine serum albumin (BSA; Jackson Immuno Research). Intense green fluorescent signals can be observed in the infected PETCs **(G–I)** as compared to the mock-inoculated **(J–L)**. **(M)** Bar graph showing the trend of integrated fluorescence intensity values (y-axis) obtained from Image J analysis of 200X magnified images representative of two experiments. The product of the mean fluorescence intensity and the percentage of area with fluorescent signal was termed integrated fluorescence intensity. **(N)** Bar graph representing normalized gene expression of PEDV *N-gene* against *EIF3K* endogenous control gene in PETCs derived from different small intestinal segments. The relative quantification values (y-axis) data obtained from ΔΔCt analysis showed an average of four replicates (Error bars = SEM) with P value < 0.001 (***) denoting statistical significance, while ns denotes no significance.

Following PEDV-inoculation, PETCs derived from the duodenum, jejunum, and ileum exhibited virus-specific CPE, such as cell rounding and detachment (**Figures 7A–C**), observed as early as 24 hpi. No CPE was observed in the mock-inoculated PETCs, with no apparent differences in CPE across regions (**Figures 7D–F**). PEDV infection was confirmed through IFA, which detected PEDV N protein expression in virus-inoculated cells (**Figures 7G–I**), while mock-inoculation showed no fluorescent labeled antigen (**Figures 7J–L**). Quantitation of Integrated Fluorescence Intensity of IFA images indicated a trend suggesting higher levels of PEDV infection in jejunum-derived PETCs, followed by ileum and duodenum-derived PETCs (**Figure 7M**). However, viral RNA extracted from cell lysates demonstrated significantly higher (P < 0.0001) levels of PEDV *N-gene* RNA in jejunum-derived PETCs compared to those from the duodenum and ileum, as determined by RT-qPCR analysis (**Figure 7N**). In contrast, the collected supernatants expressed PEDV *N-gene* without significant differences observed between PETCs derived from the three small intestine regions.

## 4 Discussion

Intestinal organoids or enteroids offer a promising alternative to conventional cell culture and animal bioassay as an infection model (39). Derived from isolated porcine crypt stem cells and cultured in Matrigel, enteroids exhibit a 3D architecture that closely mimics the small intestinal epithelium *in vivo*, encompassing a majority of its cell lineage composition (23). Enteroids can be expanded through various passages without detectable alterations in their growth patterns, morphology, or gene expression profile (18, 40). Furthermore, it is possible to generate and expand a substantial number of enteroids from the intestine of a single animal, which can then be serially cultured and cryopreserved for future use. This approach has ethical advantages, such as reducing the dependence on tissues from multiple donor pigs and enabling the establishment of large enteroid stocks for individual experimental sets, leading to a highly reproducible model. Overall, porcine enteroids have emerged as valuable infection models, demonstrating their susceptibility to various pathogens (8, 25–27, 41–47).

Previous studies reported the development of porcine enteroids in Matrigel derived from the three major regions of the small intestine, i.e., duodenum, jejunum, and ileum (8, 25), yet none of them comprehensively characterized the differences between the region-specific enteroids. Moreover, a significant challenge associated with Matrigel-cultured enteroids during infection studies is in the inward orientation of the apical surface, limiting pathogen accessibility. While techniques like microinjection and apical-out culture have been introduced to address this limitation (43), they face technical complexities and inconsistencies in achieving apical-out polarity (46). Alternatively, van der Hee et al. (32) established pig ileum enteroids on transwell inserts, allowing for luminal exposure to microorganisms and bioactive compounds. Nevertheless, a comprehensive characterization of region-specific enteroids and transwell-polarized enteroids is still lacking. Thus, this study describes the development and characterization of segment-specific enteroids from the pig small intestine in Matrigel (PEMC) and transwell inserts (PETC).

The development of porcine enteroids involves multifaceted considerations affecting cellular differentiation and stemness maintenance. Standardization of culture conditions and thorough characterization of enteroids remain challenging, with variations in culture medium formulation, cell seeding density, and culture format impacting cellular lineage or phenotype expression and stem cell abundance (48). While slight variations exist in medium composition across studies, the supernatant from the L-WRN cell line (49), containing key growth factors (Wnt3, R-Spondin, and Noggin), is most commonly used (46–48). However, full ingredient disclosure for commercial medium is often lacking (8, 25, 27). Despite the seemingly superior differentiation observed with L-WRN-conditioned medium (47), further evaluation is warranted. In addition, the use of CHIR99021 (GSK-3β inhibitor) in the culture medium promotes early budding after seeding by activating the Wnt pathway, and prevents anoikis, but prolonged exposure may compromise barrier integrity (24). Our study aligns with previous research by incorporating CHIR99021 into the culture medium for the first 48 h (46, 48). On the other hand, the inclusion of Y-27632 in the culture medium remains controversial and is typically limited to the initial days (46, 48), due to its potential adverse effect on epithelial barrier assembly (32).

The optimal number of cells for seeding onto transwell inserts in PETCs remains undefined, and the process would need to be standardized case by case. While higher seeding densities may expedite confluency and appropriate morphology for infection, they can also lead to altered cell expression profiles (48). The present study aligns with previous reports in utilizing higher cell densities (3-7 x 10^5^ cells/cm^2^), highlighting the need for uniform seeding conditions to ensure consistent outcomes (46). Additionally, variations in 2D enteroids culture methods for infection studies, such as transwell inserts (45, 46) versus plate wells (8, 25, 27), can further influence cell phenotype differentiation (48). These variations underscore the importance of standardizing culture conditions and thoroughly characterizing the model used in the study to maintain stemness and ensure reliable results in porcine enteroids cultures.

Regarding possible differences in enteroids differentiation and proliferation, Luo et al. (25) highlighted the ease of collecting duodenal crypt cells and their rapid growth into enteroids. However, as reported by Li et al. (8), we found no significant differences in the timing of crypt cell differentiation or proliferation across small intestine segments, nor in cell yield, which was rather influenced by factors like tissue sample size and sample quality. Although CellTiter Glo-3D viability assay suggested a slightly higher metabolic activity in duodenal PETCs at day 2, these findings were not statistically significant. Future studies should include multiple donor animals to address potential variability across segments.

This study aimed to characterize cell phenotypes and functional markers in porcine enteroids derived from three regions of small intestine segments using IHC and gene expression analysis. Results revealed no significant regional differences in key cellular constituents between proximal and distal enteroids, including epithelial cells, goblet cells, enteroendocrine cells, Paneth cells, proliferating cells, stem cells, and components of the epithelial barriers. Consistent with previous research, the gene expression profiles and cellular marker staining in enteroids closely resemble those observed in native intestinal tissues (50). However, the study acknowledges limitations in quantitatively assessing phenotype composition between enteroids and intestinal tissues due to challenges in processing enteroids into paraffin blocks, their inherent heterogeneity in size and composition, and variations in maker expression across continuous sections within each enteroid.

Numerous challenges were encountered during IHC staining, especially with PEMCs, which are worth discussing as a troubleshooting guide for future research due to limited available information elsewhere. Following conventional tissue processing procedures, embedding PEMCs into paraffin blocks proved challenging due to their size and background contrast in paraffin. Alternatively, PEMCs were embedded in agarose plugs, facilitating fixation and maintaining their positions during the dehydration process for paraffin embedding. Variability within enteroids and sectioning limitations were noted regarding cell marker (antigens) expression, as exemplified by inconsistent villin staining. Background interference from Matrigel required adjustments to staining conditions, including reducing incubation times from 1 h to 30 min and counterstaining duration from 30 s to 10 s. Manual deparaffinization and rehydration were preferred for intact PETCs sections to prevent damage. These challenges, alongside previous ones, emphasize the complexity of accurately quantifying positive signals for PEMCs and PETCs in IHC.

The present study includes the comparative assessment of the susceptibility of regional-specific PETCs to PEDV infection using a highly pathogenic PEDV non-S-INDEL strain. Although multiple segments of the porcine small intestine are susceptible to PEDV, higher signal for PEDV antigen has been consistently reported in the jejunum and ileum (5, 7), corresponding to increased tissue tropism observed in the mid-jejunum (51). While previous studies reported no major differences in PEDV replication between enteroids derived from the duodenum, jejunum, and ileum (8), our study revealed significantly higher fluorescence density of PEDV N protein and increased expression of PEDV *N-gene* in jejunum-derived PETCs. This apparent discrepancy across studies may be attributed to variations in viral load within the inoculum, with Li et al. (8) using an infectious dose of 10^4^ TCID_50_/mL at the end point of 72 hpi, whereas our study used a higher dose of 10^5^ TCID_50_/mL.

PEDV susceptibility and infection outcome are influenced by several factors, including virus strains, susceptible cell phenotypes, and the availability of host cell proteases within the small intestinal segments, while the functional receptor(s) for PEDV remain unknown (52, 53). The diverse compositions of cell types across different small intestine segments may contribute to the segmental tropism for PEDV (54). To date, enterocytes are recognized as the primary site of PEDV infection (52), although infection of other cell types, such as goblet cells (55) and stem cells (8) has also been reported. Goblet cells, crucial for mucosal protection against pathogens, are of particular interest. PEDV infection has been associated with a reduction in goblet cell numbers, leading to compromised mucin production and a defective mucus layer (55). Although the exact role of goblet cells in PEDV infection remains unclear, their involvement is suggested by the presence of PEDV antigen in goblet cells both *in vivo* (55) and in porcine enteroids (8). Additionally, cellular markers such as tight junction marker occludin (56) or sialic acid (53) may be associated with PEDV infection and warrant further investigation.

Overall, the use of intestinal segment-specific enteroids cultures provides a suitable high throughput platform (e.g., scalable up to 96-transwell plates) platform for virological studies, circumventing the complexities present in *in vivo* studies and the significant limitations inherent to traditional cell culture (39). Standardizing culture conditions and comprehensively characterizing the model used are imperative for advancing enteroids-based infection models and ensuring their reliability and reproducibility.

## Data Availability

The original contributions presented in the study are included in the article/supplementary material. Further inquiries can be directed to the corresponding authors.
